# *Staphylococcus aureus* Biofilms and Their Response to a Relevant *in vivo* Iron Source

**DOI:** 10.3389/fmicb.2020.509525

**Published:** 2020-12-21

**Authors:** Priscila Dauros-Singorenko, Siouxsie Wiles, Simon Swift

**Affiliations:** Department of Molecular Medicine and Pathology, Faculty of Medical and Health Sciences, University of Auckland, Auckland, New Zealand

**Keywords:** gene expression, phenol soluble modulins, ferric uptake regulator, iron-regulated surface determinant B, hemoglobin, iron, biofilm, Staphylococcus aureus

## Abstract

Biofilm infections can be chronic, life threatening and challenging to eradicate. Understanding *in vivo* stimuli affecting the biofilm cycle is one step toward targeted prevention strategies. Iron restriction by the host is a stimulus for biofilm formation for some *Staphylococcus aureus* isolates; however, in some infection scenarios bacteria are exposed to abundant amounts of hemoglobin (Hb), which *S. aureus* is able to use as iron source. Thus, we hypothesized a role for Hb in the biofilm infection. Microplate “biofilm” assays showed biofilm-matrix production was increased in the presence of hemoglobin when compared to the provision of iron as an inorganic salt. Microscopic analysis of biofilms showed that the provision of iron as hemoglobin consistently caused thicker and more structured biofilms when compared to the effect of the inorganic iron source. Iron responsive biofilm gene expression analysis showed that Agr Quorum Sensing, a known biofilm dispersal marker, was repressed with hemoglobin but induced with an equivalent amount of inorganic iron in the laboratory strain Newman. The gene expression of two biofilm structuring agents, PSMα and PSMβ, differed in the response to the iron source provided and was not correlated to hemoglobin-structured biofilms. A comparison of the model pathogen *S. aureus* Newman with local clinical isolates demonstrated that while there was a similar phenotypic biofilm response to hemoglobin, there was substantial variation in the expression of key biofilm dispersal markers, suggesting an underappreciated variation in biofilm regulome among *S. aureus* isolates and that no general inferences can be made by studying the behavior of single strains.

## Introduction

The ability to coordinate the expression of diverse virulence factors contributes to the broad range of infections caused by *Staphylococcus aureus*. Biofilms of *S. aureus* are a common virulence trait, important in the etiology of life-threatening infections such as endocarditis, central line-associated bloodstream infections, ventilator associated pneumonia, implant-related infections, and surgical site infections (Costerton et al., 1999). Biofilms are bacterial aggregations embedded in an extracellular matrix with specific gene expression or metabolic networks different from their planktonic counterparts (O’Toole et al., 2000). Biofilms provide an infectious chronic reservoir with a higher resistance to immune defenses and exogenous antibiotics, thus biofilm infections present harder challenges for eradication (Pratten et al., 2001; Otto, 2018).

The *in vitro* studies of biofilms do not, however, provide a complete model of biofilm activities in disease as all necessarily include compromises (Otto, 2013). One limitation many of these studies have made is in the medium used, with rich laboratory media providing conditions that promote biofilm formation (Boles et al., 2010; Atshan et al., 2013). A review of the effect of growth environment upon the control of virulence gene expression does not demonstrate a good correlation between the situation *in vitro* and situation in disease. Dissection of the regulation of virulence is usually made in strains growing in rich media; however, mimicking the host environment (e.g., serum or physiological media) has been shown to induce different regulatory pathways (Pragman and Schlievert, 2004; Oogai et al., 2011; Jenul and Horswill, 2018). Strains used for *in vitro* studies may also be criticized. The properties of genetic tractability, gene knockout availability and genome sequence offer considerable advantages; however, there are also disadvantages associated with reliance upon laboratory adapted strains (Fux et al., 2005).

Stimuli, directly or indirectly, enhancing biofilm formation or dispersal are diverse; and the molecular basis of the effect of these stimuli in the biofilm are far from being completely elucidated (Lister and Horswill, 2014). In *S. aureus* detachment is regulated by Agr Quorum Sensing, where cell density-dependent activation of extracellular matrix degrading enzymes is proposed to release individual cells, clusters, or large emboli from the biofilm (Boles and Horswill, 2008). Biofilm matrix composition varies among strains and growth conditions, including polysaccharides, proteins, and eDNA (Payne and Boles, 2015). Phenol Soluble Modulins (PSMs) are Agr-regulated amphipathic surfactant-like peptides with cytolytic and proinflammatory roles in infection, particularly in immune cells, however, they also have shown important structuring and dispersal roles in *S. aureus* biofilms (Peschel and Otto, 2013). These detached cells become planktonic cells and/or re-attach at remote sites allowing the persistent dissemination of the infection in the body.

Iron restriction is one the host’s antimicrobial defenses and a successful pathogen is able to overcome this challenge by acquiring iron from available iron sources (Dauros-Singorenko and Swift, 2014). However, this iron restricted environment has also been reported as an important signal for several bacterial pathways such as biofilm induction in some *S. aureus* strains, whereas the opposite dispersal effect can be seen with the provision of inorganic iron to the biofilm (Johnson et al., 2005; Haley and Skaar, 2012). Hemoglobin (Hb) is the most abundant iron source in the host (Crichton, 2001), and *S. aureus* has adapted to acquire Hb and use iron from it in an infection (Mazmanian et al., 2003; Skaar et al., 2004). *Staphylococcus aureus* incorporates hemoglobin using the Isd system, where the main hemoglobin receptor IsdB allows specific recognition of the iron source (Kim et al., 2010). Haem is internalized into the cytoplasm and then iron is released to participate in bacterial intracellular homeostasis, a process regulated by Ferric Uptake Repressor (Fur; Escolar et al., 1999; Torres et al., 2006). The investigation of the proposed essential role of IsdB in the acquisition of iron from Hb in infection has been studied with conflicting results (Torres et al., 2006; Hurd et al., 2012; Pishchany et al., 2014). Additional roles for IsdB as an adhesin that are independent of the ability to bind Hb have been proposed and IsdB also interacts directly with a platelet receptor inducing platelet aggregation in bloodstream (Miajlovic et al., 2010; Zapotoczna et al., 2013). Furthermore, haem-iron is reported to contribute to the regulation of staphylococcal virulence factors (Schmitt et al., 2012; Laakso et al., 2016; Casabona et al., 2017). Pynnonen et al. (2011) were the first to demonstrate that hemoglobin promoted *S. aureus* adhesion when investigating factors influencing nasal colonization added as supplements to a medium based upon Tryptic Soy Broth. The inhibition of Agr Quorum Sensing by hemoglobin observed in planktonic culture was proposed as an explanation for the improved colonization (Pynnonen et al., 2011). Schlievert et al. (2007), in media based on Todd-Hewitt broth, have described how the α and β chains of hemoglobin can inhibit Agr Quorum Sensing and another regulatory system (SrrA-SrrB) linked to Agr. Moreover, in the same strain, deleting *agr* in *S. aureus* biofilms grown in iron restriction has revealed a critical role of Agr in biofilm formation, previously undetected when culturing biofilms in Trypticase soy broth (TSB) media (Beenken et al., 2003; Johnson et al., 2008). It is clear that in some scenarios *S. aureus*’ exposure to abundant hemoglobin (e.g., biofilms associated with central venous catheter) could provide a new facet to the progression of a biofilm infection beyond its importance in the iron starved to iron replete transition of the infection.

To test this hypothesis, we screened *S. aureus* laboratory adapted strain Newman and clinical isolates looking to characterize the biofilm forming capacity in a physiological iron restricted medium supplemented with an *in vivo* relevant iron source (hemoglobin) and a commonly used inorganic iron salt (ferric chloride). In general, we found inorganic iron salts promoted biofilm dispersal, whereas the provision of Hb promoted biofilm formation by increasing matrix production. We discovered that hemoglobin exposure is accompanied by more structured biofilm architecture across the *S. aureus* isolates investigated. The investigation of gene expression by quantitative reverse transcription PCR (RT-qPCR) in biofilm populations demonstrated cells were iron-restricted in the absence of iron and gave appropriate predicted responses to the provision of inorganic iron salts and hemoglobin. The investigation of the divergent phenotypic effects of these two iron sources, as a single stimulus, upon *S. aureus* biofilms identified a role for Agr Quorum Sensing in Newman that was not strongly correlated to the expression of two PSMs and a variation in the aforementioned genes among isolates in response to the same single iron stimulus.

## Materials and Methods

### Bacterial Strains and Growth Conditions

Throughout this study, *Staphylococcus aureus* strain Newman (ATCC 25904; Duthie and Lorenz, 1952; Johnson et al., 2005) and clinical isolates from bacteremic infection (BC03) and from endocarditis infection (EC12) obtained from The Auckland Hospital (Auckland, New Zealand) were used (Supplementary Table S1). For cloning experiments *Escherichia coli* TOP10 and DB3.1 (both Thermo Fisher Scientific) were used. Routine bacterial culture was in Difco TSB or on Difco Trypticase Soy agar (TSB and TSA; Fort Richard Laboratories, Auckland, New Zealand). Before each experiment bacterial cells were recovered from −80°C stocks onto Horse Blood agar (Fort Richard Laboratories) and then transferred into 10 ml of physiological iron restricted RPMI 1640 medium (Thermo Fisher Scientific) in a V-bottomed 50 ml polystyrene tube and allowed to grow overnight at 37°C with shaking at 200 rpm to minimize evolved subcultures (Fux et al., 2005) and to mimic aspects of the *in vivo* environment. When specified in the assay, growth media was supplemented either with iron III chloride (FeCl_3_, Acros Organics) at a final concentration of 2 or 40 μM; or hemoglobin (Hb; Sigma) at a final concentration of 0.5 or 10 μM. Iron concentrations were selected based on published studies showing full (10–100 μM Fe^3+^) or partial (<10 μM Fe^3+^) de-repression of Fur (Johnson et al., 2005, 2011). Antibiotic selection was used when specified at concentrations of chloramphenicol (Cm; Sigma) at 7 μg ml^−1^ or ampicillin (Amp; Sigma) at 100 μg ml^−1^. Anhydrotetracycline (aTc; Sigma) was added to media at 500 ng ml^−1^.

### Biofilm Quantification by Microplate Assay

A microplate model was used to determine biofilm formation capacity in different growth media. For matrix quantification assays, a RPMI 1640 overnight culture was centrifuged at 5000 × *g* for 10 min, resuspended in fresh medium and diluted to a final concentration of 1 × 10^7^ CFU ml^−1^ in 200 μl of desired medium (RPMI 1640 or RPMI 1640 supplemented with 2 and 40 μM of FeCl_3_ or 0.5 and 10 μM hemoglobin) in each well of a sterile 96-well microplate (Sarstedt, Germany). The microplate was incubated at 37°C and 200 rpm for 24 h or the stated time frame. Spent medium was removed from each well and washed twice with Phosphate Buffered Saline (PBS, Sigma-Aldrich) before matrix quantification. A protocol described by Toté et al. (2008) was used to measure the biofilm matrix. Dimethyl methylene blue (DMMB; Sigma) is a dye specific to stain the main biofilm matrix component Polysaccharide Intercellular Adhesin (PIA) in *S. aureus*. DMMB absorbance was measured at 650 nm using an EnSpire plate reader (Perkin Elmer). Resazurin stain (Sigma) was used to assess the viability of the biofilms according to protocol described by Toté et al. (2008). Absorbance and fluorescence measurements from media alone were subtracted from biofilm measurements. Data analysis was performed from four replicates (wells in the 96-well plate) and three independent experiments. Normal distribution was confirmed by D’Agostino Pearson test with software GraphPad Prism (version 5.02). Statistical differences were determined by two-way ANOVA and Bonferroni post-test. Statistical tests and graphic representations were done with GraphPad Prism software (version 5.02).

### Construction of *fur* Mutant

To study the role of Fur in the biofilm response to inorganic iron and hemoglobin, a *fur* mutant was made in *S. aureus* Newman. PCRs were performed using Phusion Flash High-Fidelity PCR Master Mix (Thermo Fisher Scientific) according to the manufacturer’s instructions and primers sequences are shown in Supplementary Table S1. PCR amplifications of 1 kb DNA fragments of flanking upstream (primers Fur-up-F-attB2 and Fur-up-R-sacII) and downstream (primers Fur-down-F-sacII and Fur-down-R-attB1) regions of *fur* were made from *S. aureus* Newman chromosome. Purified DNA flanking fragments were digested with *Sac*II (New England Biolabs) and ligated with T4 DNA Ligase (New England Biolabs). PCR with Fur-up-F-attB2 and Fur-down-R-attB1 primers was set to amplify the whole 2 kb fragment, using the ligation product as template. Gateway Technology (Thermo Scientific Scientific) was used to clone the 2 kb PCR product into pKOR1 (Bae and Schneewind, 2006) resulting in plasmid pFK. pFK was transformed into *E. coli* TOP10 competent cells with selection on ampicillin at 28°C. Purified pFK was then electroporated into *S. aureus* RN4220 (Kreiswirth et al., 1983) with selection on Cm at 28°C (Kraemer and Iandolo, 1990; Schenk and Laddaga, 1992). pFK plasmids from RN4220 were transduced into *S. aureus* Newman with phage Ø11 (Novick, 1991) selecting for Cm resistance at 28°C. The allelic replacement was initiated by growing the successful *S. aureus* Newman transductants in broth at 42°C to inhibit plasmid replication then streaked to TSA containing Cm and incubated at 42°C for 24 h to select cells with insertion of the plasmid into the chromosome. A single colony was grown overnight in TSB without antibiotic selection at 28°C and plated out on TSA-aTc and incubated at 37°C overnight allowing excision of the plasmid from the chromosome, with aTc inducing killing of cells still carrying the vector (Bae and Schneewind, 2006). The successful construction of *S. aureus* Newman *∆fur* mutants was confirmed in several ways: mutants lacked Cm resistance on agar plates; PCR amplification (Fur-down-R-attB1 and Fur-up-F-attB2) of mutant chromosomal DNA amplified the 2 kb region excluding *fur*, not the parental 2400 bp product; lack of response of known iron/Fur regulated cell wall proteins was checked by discontinuous 12% SDS PAGE (Supplementary Figure S1); and finally, the absence of amplification products using *fur* specific primers (Fur-F and Fur-R) with RT-qPCR (Supplementary Figure S1).

### Biofilm Structure by Confocal Microscopy

To determine the architecture of biofilms grown with two iron sources, the biofilm structure was microscopically analyzed. Biofilms were grown as described for biofilm quantification by microplate assay, scaled up to a 24-well plate (Falcon, United States) containing 1 ml of medium. After 24 h, spent medium was removed from each well and washed twice with PBS before following samples preparation for microscopy. A 150 μl mixture containing 147 μl of water, 1.5 μl of Syto9 for live cells (3.34 mM) and 1.5 μl of Propidium Iodide for dead cells (20 mM) was added to each well containing biofilms. The plate was incubated at RT and protected from light for 30 min, following the manufacturer’s instructions (LIVE/DEAD BacLight Bacterial Viability Kit, Molecular Probes, Inc.). Confocal laser scanning microscopy (CLSM) was performed using LSM 710 inverted microscope (Carl Zeiss, Germany). Images were acquired using a LD Plan-Neofluar 40x/0.6 Korr M27 objective focusing from bottom on the biofilms in the multi-well plate. Z-stacks were obtained at 2.5 μm intervals with single image area of 212.36 × 212.36 μm. ZEN 2011 software was used for image acquisition and processing. Biofilm biomass, average thickness and roughness were analyzed with COMSTAT 2 software (Heydorn et al., 2000). Data analysis was performed from duplicates (two field views in each well) and six wells from different experiments. Normal distribution was confirmed by D’Agostino Pearson test with software GraphPad Prism (version 5.02). Statistical significance was assessed by one-way ANOVA and Tukey post-test. Statistical tests and graphic representations were done with software GraphPad Prism (version 5.02).

### RNA Extraction From Planktonic Cells and Biofilms

Biofilm cells were quickly scraped off from the bottom of the petri dish with sterile 1 ml pipette tips to collect them in a 50 ml polystyrene tube. RNA extraction was performed using an Ambion RiboPure-Bacteria Kit (Thermo Fisher Scientific). All consumables used through this method were certified RNAse free. Briefly, biofilm cells were collected by centrifugation and cells were re-suspended in 1 ml of Ambion RNAlater RNA stabilizing solution (Thermo Fisher Scientific) and kept at 4°C until further use. RNAlater removal was done following the manufacturer’s instructions. Ambicin L lysostaphin (AMBI Inc., New York, United States) 17.5 μl (20 mg ml^−1^) and 350 μl of RNAWIZ solution were added to the Zirconia Beads tube and incubated at 37°C for 10 min before vortexing in a Beads Ruptor 24 Homogenizer (Omni International, United States) for 5 min at a speed of 3.25 ms^−1^. The rest of the protocol was performed following the manufacturer’s recommendations. Planktonic cells from 10 ml of iron-treated cultures grown in 50 ml polystyrene tubes were collected by centrifugation and continued with same RNA extraction protocol as biofilm samples. The purity of RNA samples was screened with Nanodrop 2000 (Thermo Fisher Scientific). RNA samples with ratios A260\280 nm (estimating protein contamination) and A260\230 nm (estimating phenol, carbohydrates or salt contamination) above 2.0 were included in the study. The integrity of RNA was screened with Experion™ Automated Electrophoresis System (Bio-Rad). Samples were prepared using Experion™ RNA StdSens Analysis Kit (Bio-Rad) and following the manufacturer’s recommendations. RNA electropherograms were inspected for RNA fragmentation. RNA meeting the criteria of two distinctive peaks of 23S and 16S, no noise or defined peaks between, after or before 23S and 16S peaks were judged as unfragmented and further used for expression studies.

### Gene Expression by RT-qPCR

Primers used in RT-qPCR are listed in Supplementary Table S1. Power SYBR® Green RNA-to-C_T_™ 1 Step Kit was used in the qPCR reactions, and 10 μl reactions were made according to following details: Power SYBR® Green RT-PCR Mix (2X) 5 μl, Forward Primer (10 μM) 0.3 μl, Reverse Primer (10 μM) 0.3 μl, RT Enzyme Mix (125X) 0.08 μl, RNA Template (10 ng final concentration) 4.32 μl. The mix was loaded into a 384-well plate (MicroAmp Optical 384-well Reaction plate, Applied Biosystems), and placed into ABI 7900HT Fast Real Time PCR System (Applied Biosystems). Running conditions: one holding stage of 30 min at 48°C (reverse transcription), one holding stage of 10 min at 95°C (for DNA Polymerase activation), 40 cycles of 15 s at 95°C (denature) and 1 min at 60°C (anneal/extend). For dissociation curve purposes an extra stage was added: 15 s at 95°C (denature), 15 s at 60°C (anneal), and 15 s at 95°C (denature).

### Strain Typing

Agr type, of our own collection of endocarditis and bacteraemia isolates, was determined by PCR and specific primers (Supplementary Table S1) for each Agr type as described by Strommenger et al. (2004).

### Data Analysis

qPCR data collection and visualization was performed by software SDS v2.4 (Applied Biosystems). For calculation of PCR Efficiency of the qPCR reactions free online software LinRegPCR was used (Ramakers et al., 2003). For gene expression analysis licensed software qbasePLUS (Biogazelle; Hellemans et al., 2007) was used. Gene expression was normalized to five reference genes: 16S, *gyrB*, *pta*, *tpi*, and *hu* (Valihrach and Demnerova, 2012). Data of relative gene expression ratios was obtained from triplicates in each RNA extraction from the biofilm and three independent experiments. Relative expression ratios were transformed into square root (SQRT) values. Statistical significance was determined by Kruskal Wallis test and Dunn’s post-test. Statistical tests and graphic representations were done with software GraphPad Prism (version 5.02).

## Results

### Hemoglobin Promotes *Staphylococcus aureus* Biofilm Matrix Production

**Figure 1:**
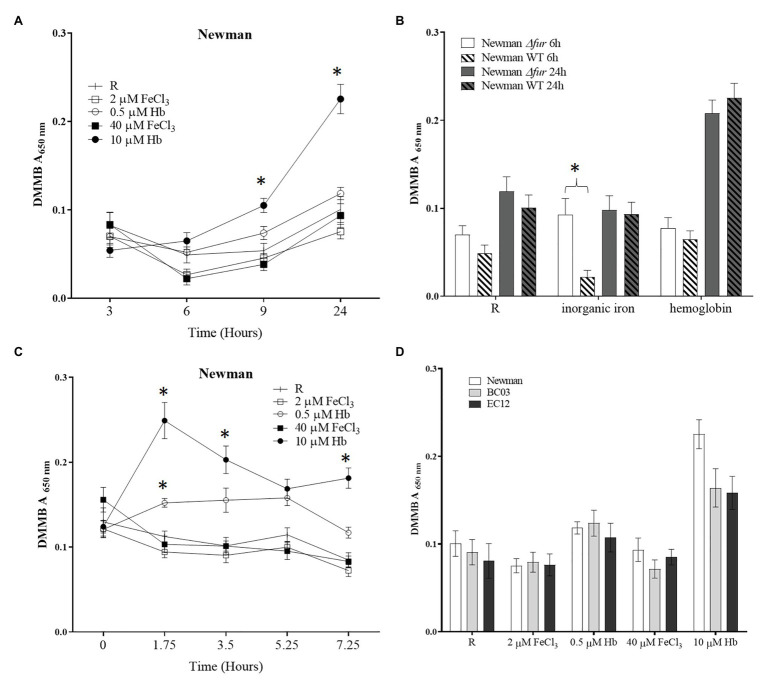
Biofilm matrix quantification with different iron sources. A *Staphylococcus aureus* culture grown in RPMI overnight resuspended in fresh medium was diluted to a final concentration of 1 × 10^7^ CFU ml^−1^ in 200 μl of medium [RPMI or RPMI supplemented with 2 and 40 μM of FeCl_3_ or 0.5 and 10 μM hemoglobin (Hb)] a sterile 96-well microtiter plate. The microplate was incubated at 37°C and 200 rpm for desired time. Spent medium was removed and biofilms were washed twice with Phosphate Buffered Saline (PBS). Biofilm matrix was stained with dimethyl methylene blue (DMMB). (A), biofilm formation through time in Newman WT; (B), biofilm formation in Newman *∆fur*; (C), dispersing biofilm through time with addition of iron source in Newman WT; and (D), biofilm matrix level at 24 h in Newman and two clinical isolates, BC03 (bacteraemia) and EC12 (endocarditis). Data is plotted as mean and error bars represented by SEM. Measurements were taken from quadruplicates wells in a microplate in three independent experiments. Stars denote significant difference (*p* < 0.05) tested by one-way ANOVA and Tukey post-test.

To investigate the initial iron-dependent biofilm attachment response to a surface, a microplate assay was performed to specifically quantify the main matrix component PIA in biofilms. Biofilms were grown in physiological media RPMI 1640 and the presence of two concentrations of FeCl_3_ or hemoglobin. The model laboratory strain *S. aureus* Newman displayed a significant decrease in biofilm matrix levels when cultured with low and high inorganic iron concentrations (Figure 1A), consistent with other studies showing that *S. aureus* Newman biofilms are inhibited by iron (Johnson et al., 2005). On the other hand, culture with hemoglobin led to 200% more biofilm matrix at 24 h of incubation than the inorganic iron source in *S. aureus* Newman (Figure 1A). Neither iron source nor concentration triggered a significant change in biofilm viability assayed by resazurin (Supplementary Figure S2). These results suggest that a first encounter with hemoglobin might be sensed as a signal to quickly induce biofilm formation by matrix production in *S. aureus* Newman.

To determine if this differential response to iron sources was regulated by the iron uptake regulator Fur in *S. aureus* Newman, a *fur* deletion mutant was created and subjected to the same assay. The response to inorganic iron was not apparent in the *fur* mutant after 6 h (Figure 1B), suggesting that Fur may repress some aspect of matrix formation at later stages and plays a more evident role in early stages of biofilm formation. The positive effect on biofilm formation promoted by Hb was not affected by *fur* mutation suggesting that this biofilm response is independent of iron homeostasis (Figure 1B).

To determine if the differential response to iron sources is linked to the attachment phase of biofilm formation, a “dispersal” assay was performed. A 24 h biofilm was grown in iron-restricted medium and then given the iron sources indicated as supplements. Effects on biofilm matrix (DMMB assay) and viability (resazurin assay) were measured through time at 1.75, 3.5, 5.25, and 7.25 h after the addition of iron sources. The provision of hemoglobin led to a dose dependent increase in biofilm matrix (Figure 1C). There was no effect on viability.

To expand upon the limited predictive value of experiments with a single laboratory-adapted strain, clinical isolates from infections where bacterial cells are exposed to abundant hemoglobin were assayed, showing the same trend i.e., increased biofilm matrix in the presence of hemoglobin (Figure 1D). Taken together, these results suggest that the opposing biofilm responses to the iron sources investigated are independent of the iron homeostasis, are not limited to the early events in biofilm formation and are common across a number of clinical strains.

### Hemoglobin Promotes a Structured Biofilm in *Staphylococcus aureus*

**Figure 2:**
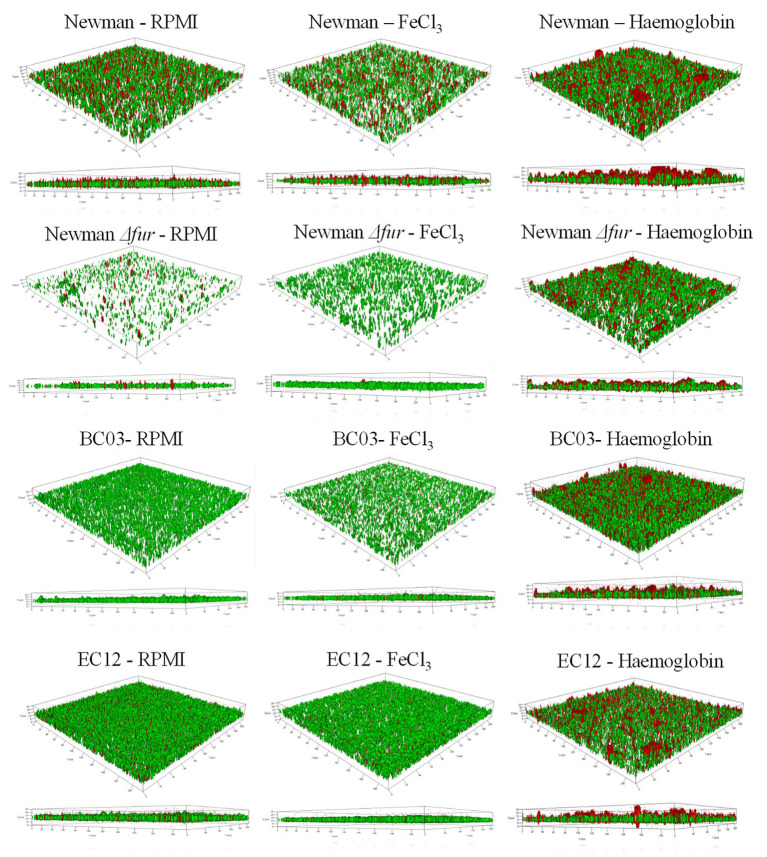
Representative CLSM Z-stacks of *S. aureus* biofilms grown under three iron treatments. An iron restricted overnight culture was resuspended in fresh medium and diluted to a final concentration of 1 × 10^7^ CFU ml^−1^ in 1 ml of medium (RPMI, 40 μM of FeCl_3_ or 10 μM Hb) in a 24-well plate (BD Biosciences). The multi-well plate was incubated for 24 h at 37°C in a static incubator. Spent medium was removed from each well, and biofilms were carefully washed twice with 1 ml of PBS before samples preparation for microscopy. Syto 9 (green) represents live cells and Propidium iodide (red) represents dead cells. 3D images were made with ZEN 2010.

To further characterize the “thicker” biofilm growth obtained by culturing in the presence of Hb, CLSM was used to investigate the architecture of biofilms grown under static conditions in iron-restriction or provided with iron as FeCl_3_ or Hb. CLSM data (Table 1; Figure 2) identifies a similar general trend among *S. aureus* Newman and the clinical isolates tested. In the presence of inorganic iron, biofilms contained fewer cells and were flatter compared to a biofilm grown under iron restriction, as represented by the biofilm biomass and thickness estimations (Table 1). Culture with Hb significantly increased biofilm biomass and thickness (Table 1), which appeared more structured (Figure 2) when compared to biofilms grown with the inorganic iron source.

**Table 1:** Biofilm features estimated by Comstat2. Data is shown as mean and SD from two images of four independent biofilms. Bold numbers denote a significant difference (*p* < 0.05) tested by one-way ANOVA and Tukey post-test between iron treatments.

			*R*	FeCl_3_	Hemoglobin
Newman	Biomass (μm^3^/μm^2^)	Live	13.14 ± 1.75	9.8 ± 1.72	**15.08 ± 2.08**
		Dead	12.77 ± 1.77	10.18 ± 1.83	**13.99 ± 1.44**
	Thickness (μm)	Live	19.32 ± 2.74	13.6 ± 2.01	**18.15 ± 2.03**
		Dead	21.28 ± 3.57	16.2 ± 2.19	**20.09 ± 2**
	Roughness	Live	0.178 ± 0.04	0.338 ± 0.12	**0.158 ± 0.05**
		Dead	0.162 ± 0.03	0.216 ± 0.08	0.168 ± 0.05
Newman *Δfur*	Biomass (μm^3^/μm^2^)	Live	13.96 ± 1.63	10.02 ± 2.86	**16.77 ± 1.28**
		Dead	7.82 ± 2.07	2.68 ± 1.87	**15.7 ± 2.1**
	Thickness (μm)	Live	19.5 ± 2.5	16.26 ± 3.15	**20.66 ± 1.87**
		Dead	16.08 ± 3.53	5.6 ± 3.62	**22.15 ± 0.04**
	Roughness	Live	0.131 ± 0.02	0.323 ± 0.13	**0.151 ± 0.02**
		Dead	0.386 ± 0.13	1.34 ± 0.39	**0.176 ± 0.04**
BC03	Biomass (μm^3^/μm^2^)	Live	15.64 ± 5.37	10.6 ± 3.99	**17.249 ± 4.6**
		Dead	9.69 ± 3.73	5.45 ± 2.4	14.022 ± 4.41
	Thickness (μm)	Live	18.9 ± 3.87	14.37 ± 4.43	**21.63 ± 4.30**
		Dead	17.97 ± 2.68	10.98 ± 4.4	**23.56 ± 4.55**
	Roughness	Live	0.155 ± 0.15	0.368 ± 0.29	0.163 ± 0.10
		Dead	0.179 ± 0.06	0.633 ± 0.30	**0.166 ± 0.05**
EC12	Biomass (μm^3^/μm^2^)	Live	17.23 ± 3.08	13.756 ± 2.2	**15.75 ± 1.91**
		Dead	11.66 ± 2.31	9.15 ± 2.15	**14.018 ± 2.34**
	Thickness (μm)	Live	21.64 ± 2.23	18.51 ± 3	17.78 ± 1.93
		Dead	19.62 ± 2.21	17.63 ± 2.8	20.137 ± 4.22
	Roughness	Live	0.099 ± 0.03	0.188 ± 0.05	**0.136 ± 0.04**
		Dead	0.174 ± 0.05	0.294 ± 0.13	**0.172 ± 0.04**

Fur did not seem to play a role in the biofilm structure associated with the response to culture with hemoglobin, given that *S. aureus* Newman *Δfur* biofilms maintained the architectural features seen in the parental strain when cultured in the presence of hemoglobin. However, *S. aureus* Newman *Δfur* biofilms showed an unexpected significantly decreased amount of dead cells linked only to the presence of inorganic iron (Table 1; Figure 2), suggesting that Fur might have a role in autolysis, possibly leading to eDNA release as part of the biofilm matrix in an iron limited environment (Mann et al., 2009).

Clinical isolates showed similar trends (Table 1; Figure 2) in their biofilm architecture with the iron conditions tested when compared to *S. aureus* Newman, indicative of a conserved biofilm phenotypic response among *S. aureus* strains when exposed to hemoglobin.

### Hemoglobin Represses the Expression of the Agr QS System

**Figure 3:**
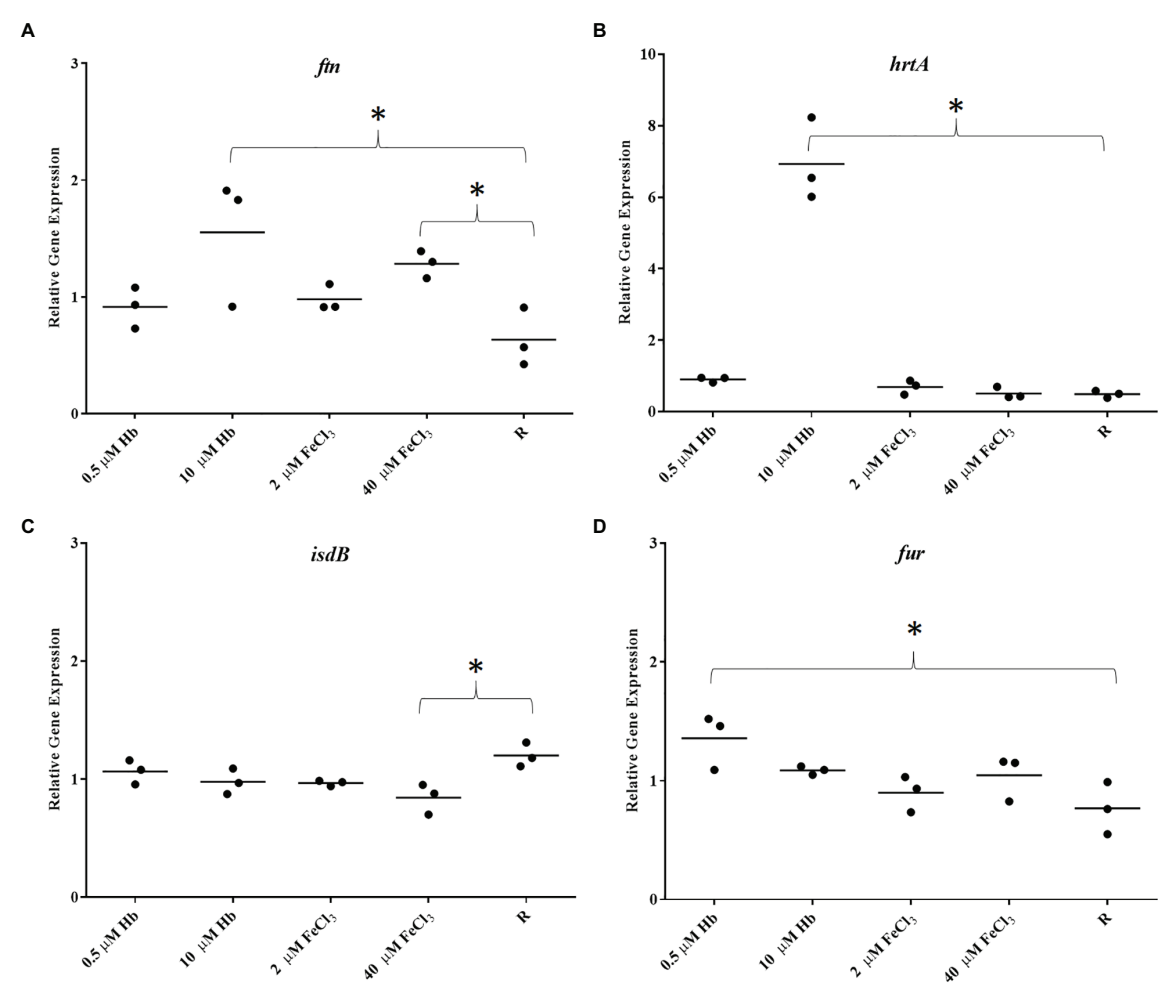
Gene expression in biofilms of Newman. **(A)**, *ftn*; **(B)**, *hrtA*; **(C)**, *isdB*; and **(D)**, *fur*. Data is plotted as mean of three biological replicates each including technical triplicates. Stars denote significant differences (*p* < 0.05) analyzed by one-way ANOVA, Kruskall-Wallis post-test.

To gain insights into biofilm gene expression in response to hemoglobin, RT-qPCR was performed on RNA extracted from 24 h biofilms and broth-cultured *S. aureus* strains grown under iron restriction or provided with inorganic iron or Hb (Figure 3).

**Figure 4:**
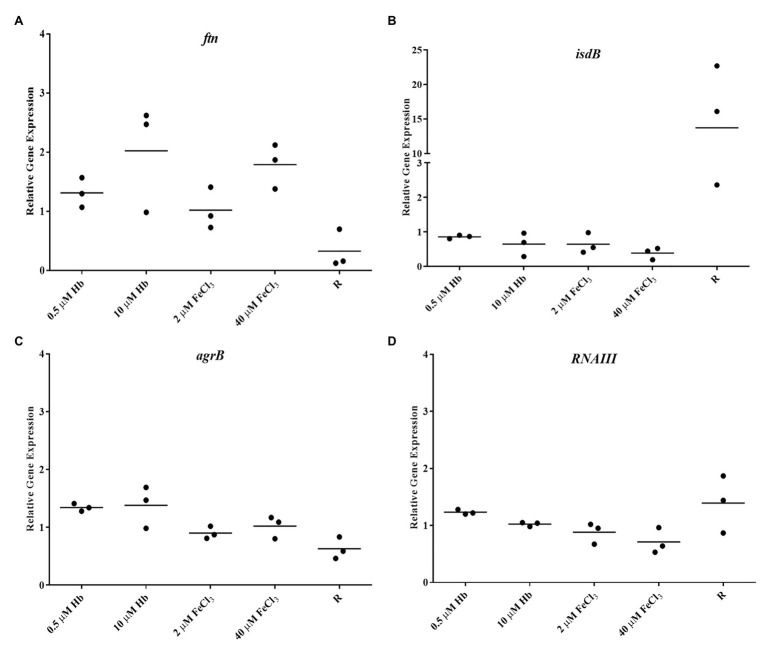
Gene expression in Newman planktonic cells. **(A)**, *ftn*; **(B)**, *isdB*; **(C)**, *agrB*; and **(D)**, *RNAIII*. Data is plotted as mean of three biological replicates each including technical triplicates. Stars denote significant differences (*p* < 0.05) analyzed by one-way ANOVA, Kruskall-Wallis post-test.

To determine if biofilms are iron sufficient or iron starved two marker genes were analyzed, *ftn* (iron storage protein ferritin; Morrissey et al., 2004) and *isdB* (main hemoglobin receptor; Torres et al., 2006), respectively. *Staphylococcus aureus* Newman biofilms showed a dose dependent increased in *ftn* expression with the provision of both iron sources when compared to iron restriction, consistent with what would be predicted for iron starved and increasingly iron-replete cells (Figure 3A). The provision of iron also repressed *isdB* expression, however, this was unexpectedly poor (Figure 3C). To confirm that haem is acquired by the biofilm, the expression of HrtA, a haem efflux pump upregulated in response to excess haem/hemin (Torres et al., 2007), was also determined. As expected *hrtA* expression in the biofilm was increased only with high Hb concentrations. The expression of *fur*, as a global regulator of gene expression in *S. aureus* responding to iron availability, was significantly upregulated with 0.5 M Hb (Figure 3D). Planktonic cells on the other hand, showed a strong *isdB* repression under all iron treatments (Figure 4). These results confirm firstly that *ftn* and *isdB* display very different iron regulation mechanisms, where *isdB* is particularly influenced during planktonic growth, and secondly both iron sources are able to penetrate the biofilm, reach the cells within, and participate in iron homeostasis.

**Figure 5:**
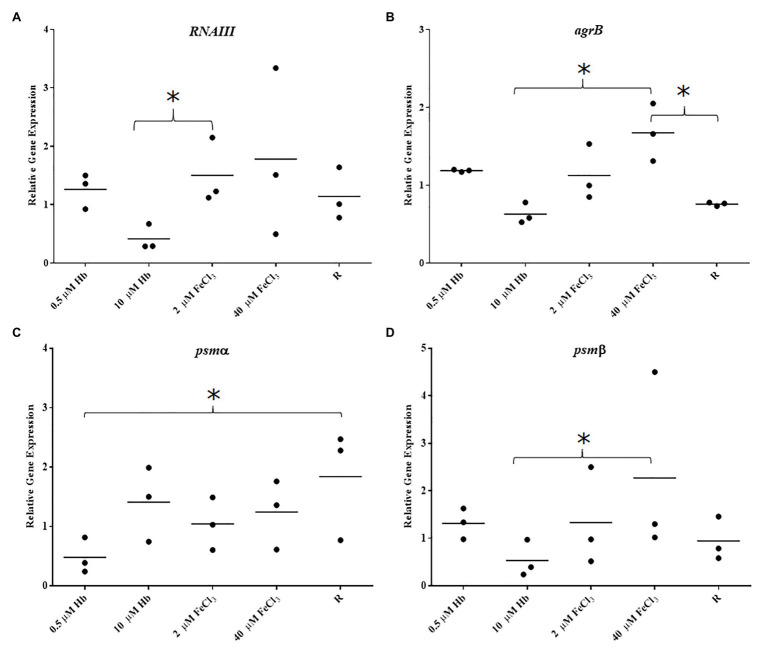
Gene expression in biofilms of Newman. **(A)**, *RNAIII*; **(B)**, *agrB*; **(C)**, *psmα*; and **(D)**, *psmβ*. Data is plotted as mean of three biological replicates each including technical triplicates. Stars denote significant differences (*p* < 0.05) analyzed by one-way ANOVA, Kruskall-Wallis post-test.

The Agr Quorum Sensing System is described as the main regulator for biofilm dispersal in *S. aureus* (Boles and Horswill, 2008). To provide some genetic context for observations made in microplate assays and CLSM, the expression of *agrB* from Agr promoter P2 and *RNAIII* from Agr promoter P3 (part of Agr QS system) was assessed. *Staphylococcus aureus* Newman biofilm populations showed an upregulation of *agrB* and *RNAIII* with inorganic iron and a downregulation with hemoglobin, with a significant difference between the responses to these iron treatments (Figures 5A,B). Once again, this response was specific to the biofilm, given that planktonic cells showed a different iron responsive pattern (Figures 4C,D), results corroborated by other groups, finding addition of inorganic iron caused extensive repression of RNAIII in planktonic cells of *S. aureus* grown in complete iron-depleted medium (Oogai et al., 2011). These results suggest that reduced Agr activity is contributing to form thicker biofilms specifically in the presence of hemoglobin as the main iron source, since adhesins are upregulated in favor of cell attachment (Novick and Geisinger, 2008) and dispersal processes are downregulated (Boles and Horswill, 2008; Peschel and Otto, 2013).

To correlate structured biofilms seen with hemoglobin as iron source by CLSM, expression of PSMs was investigated as these have been shown to be important in defining the architecture of *S. aureus* biofilms (Periasamy et al., 2012). The expression of *psmα* was slightly decreased with all iron sources compared to iron restriction in *S. aureus* Newman (Figure 5C). However, *psmβ* showed an iron responsive expression similar to the Agr QS i.e., upregulation with FeCl_3_ and downregulation with hemoglobin (Figure 5D). These transcriptional results suggest that each of the groups of PSM studied here are differentially regulated by iron and the overall expression of neither of PSMs correlates with structured biofilms grown with hemoglobin, as we would expect to see increased expression. A more detailed study that investigates the production and fate of the PSM peptides in the biofilm may provide further insight.

### Isolates of *Staphylococcus aureus* Have Different Gene Expression Responses to Iron Sources

**Figure 6:**
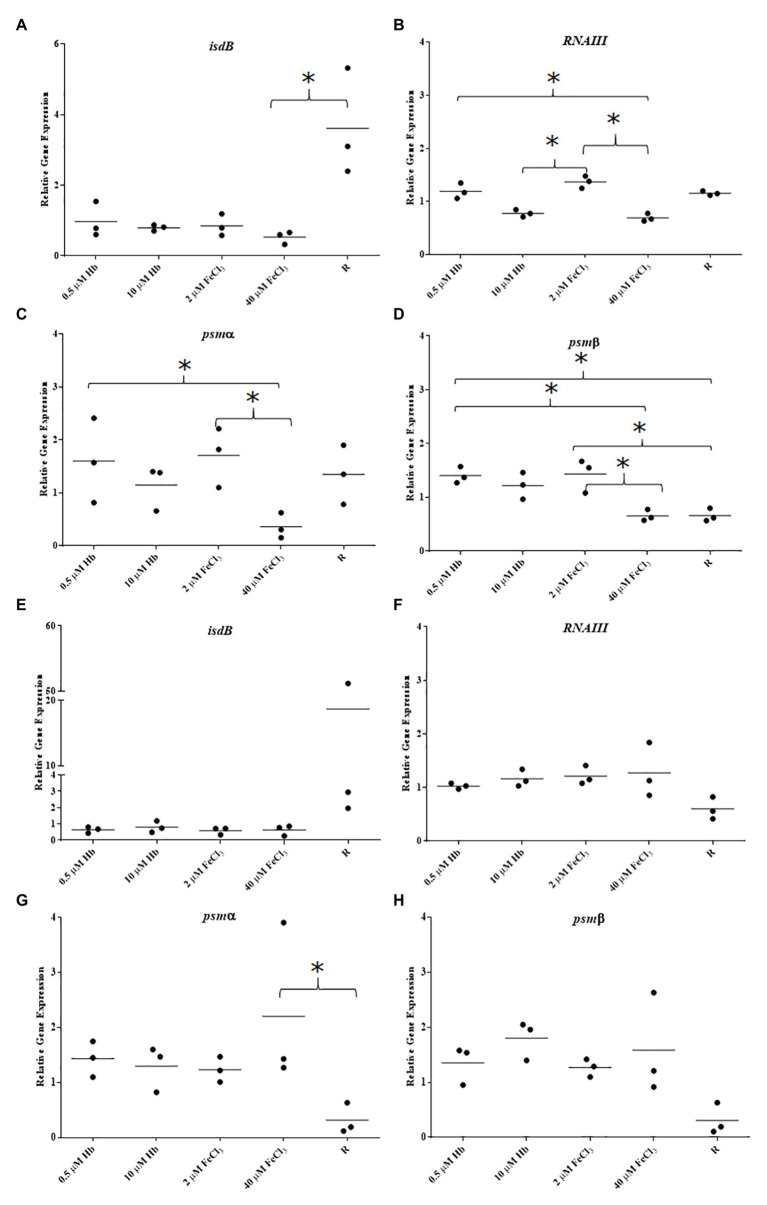
Gene expression in biofilms of clinical isolates BC03 and EC12. **(A)**, BC03 *isdB*; **(B)**, BC03 *RNAIII*; **(C)**, BC03 *psmα*; **(D)**, BC03 *psmβ*; **(E)**, EC12 *isdB*; **(F)**, EC12 *RNAIII*; **(G)**, EC12 *psmα*; and **(H)**, EC12 *psmβ*. Data is plotted as mean of three biological replicates each including technical triplicates. Stars denote significant differences (*p* < 0.05) analyzed by one-way ANOVA, Kruskall-Wallis post-test.

To determine if the response of *S. aureus* Newman biofilm gene expression to iron sources is a generalized trend among *S. aureus* isolates, biofilms from clinical isolates were analyzed for their gene expression (Figure 6). *Staphylococcus aureus* BC03 and *S. aureus* EC12 showed a pronounced Fur-dependent *isdB* repression with both iron sources (Figures 6A,E) that was more consistent with predictions based upon the literature than our observations for *S. aureus* Newman. Surprisingly, the expression pattern of *RNAIII* in response to the different iron sources varied when compared to Newman and between the clinical isolates, *RNAIII* was downregulated with both iron sources in *S. aureus* BC03 (Figure 6B) and upregulated with both iron sources in *S. aureus* EC12 (Figure 6F). Agr typing of these isolates revealed EC12 is Agr type I and BC03 is Agr type II (Supplementary Figure S4), and Newman is previously known to be Agr type I, which alone does not explain the differences in gene expression. Iron responsive gene expression of each of tested PSM was different to each other in *S. aureus* BC03 (Figures 6C,D), although expression of *psmα* and *psmβ* in *S. aureus* EC12 was similar (Figures 6G,H). These results suggest that *isdB*, Agr QS, and PSMs are highly variable in their level of expression and overall iron response among *S. aureus* isolates.

## Discussion

Iron is an important micronutrient for bacteria, essential as a cofactor for key enzymes (Sepúlveda et al., 2018). In our body, iron is sequestered as an antibacterial measure, and during an infection innate immune responses attempt to restrict iron further (Carver, 2018). Iron starvation has a profound effect on bacterial gene expression, often triggering a more virulent phenotype aimed at acquiring more iron (Ratledge and Dover, 2000; Troxell and Hassan, 2013). Successful bacterial infections are dependent on cells acquiring iron from selected, or all of the different, iron sources available in the local environment. The siderophores secreted by bacteria mainly scavenge iron in the ferric form (Fe^3+^) and hemoglobin uptake provides iron in the ferrous form (Fe^2+^; Dauros-Singorenko and Swift, 2014), however, bacterial ferric iron reductases readily convert Fe^3+^ to Fe^2+^, with Fe^2+^ acting as the ligand for Fur (Schröder et al., 2003). In this study, we asked whether the nature of the iron source influenced the responses of bacterial biofilms grown in iron restriction, and specifically whether there was more to the response to hemoglobin, a biologically relevant source of iron, when compared to an inorganic iron source. We found that FeCl_3_ and hemoglobin are distinctive stimuli to *S. aureus*, with the response to hemoglobin inducing PIA-based biofilm matrix production and a more structured biofilm, possibly through, or with the help of, the repression of the Agr QS system.

In planktonic growth, excess exogenous haem is directed to the membrane, mainly to participate as a cofactor of enzymes like cytochromes involved in energy production and ultimately may influence growth rate (Reniere et al., 2007). If membrane-directed haem does not contribute to the intracellular free iron pool, it is possible to reason that during biofilm formation with hemoglobin as main iron source, the cells are closer to being iron restricted than iron sufficient, and therefore favor biofilm formation. However, this explanation would lead to similar levels of biofilm matrix in iron restricted and hemoglobin media, which we do not see; rather we see significantly increased biofilm matrix levels with hemoglobin present compared to when conditions are iron restricted. The presence of hemoglobin in nasal secretions is the most relevant factor promoting adherence in microplate and flow cell assays with *S. aureus* SH100 grown in TSB media, whereas haem or apohaemoglobin did not cause same effect (Pynnonen et al., 2011). In our study, with physiological iron restricted media mimicking an *in vivo* environment and iron-starved cells, hemoglobin strongly promoted cell adherence or biofilm formation onto uncoated inert surfaces for a laboratory-adapted strain Newman and two other different *S. aureus* clinical strains (BC03 and EC12).

Deletion of *fur* had no influence on the biofilm induced by hemoglobin, thus, any hemoglobin effect on biofilm formation does not go through the classical iron homeostasis mechanisms in *S. aureus* Newman. Fur has been reported with a negative or repressor role in low-iron biofilm formation at the initial stages of adhesion but a positive role in the later stages of biofilm formation in *S. aureus* Newman (Johnson et al., 2005). In this study, Fur did not have a significant role at initial adhesion or later stages of biofilm formation in low iron conditions (RPMI 1640 media), which might be attributable to the RPMI 1640 media used here not being fully iron depleted. Studies that have noted an effect (Johnson et al., 2008) have used RPMI 1640 that has been further depleted of metal ions by an additional treatment with chelators. We chose not to further deplete the basal formulation by chelation, considering the need to supplement chelated medium with 10% RPMI 1640 to replace trace metal ions that would also have been removed as the introduction of an additional unnecessary variable. We conclude that the effect of hemoglobin inducing biofilm formation is independent of its function as iron source.

Confocal laser scanning microscopy images further support that hemoglobin induces different biofilm formation. For unbiased comparative purposes, the software Comstat was originally developed to analyze flow cell biofilms of *Pseudomonas* spp. and quantifies their structural changes during a time frame of 10 days (Heydorn et al., 2000). Parameters of biofilm biomass and thickness were significantly increased in biofilms exposed to hemoglobin compared to biofilms exposed to inorganic iron. The roughness coefficient has been reported as a representation of the structure of the biofilm; however, in the experimental conditions used in this assay (24 h static biofilms) this coefficient demonstrated a lack of representation of observational data. As observed in 3D CLSM images, biofilms grown with hemoglobin presented relatively more “structured” features, which is of importance and can determine the fate of the biofilm infection. Certain biofilm topologies can be more susceptible to the host immune system cells (Leid et al., 2002; Günther et al., 2009), detachment by mechanical forces or matrix breaking enzymes (Flemming and Wingender, 2010), and resistance to antibiotics (Olsen, 2015). Isolation of Viable But Not Culturable (VBNC) variants of *Staphylococcus epidermidis* and *S. aureus* from biofilms associated with 77% of explanted Central Venous Catheters (CVC) suggest biofilms are a niche for adaptation to VBNC variants, indicative of an unexplored connection between the blood stimulus and the rise of these variants (Zandri et al., 2012). To our best knowledge, architecture of *S. aureus* biofilms grown iron restricted conditions (RPMI media), assessed by CSLM has not been reported before.

Biofilms of *S. aureus* Newman grown with hemoglobin presented similar live/dead cells biomass ratio compared to iron restricted grown ones, confirming that 10 μM of hemoglobin is not a stress with a substantial toxicity load to the biofilm leading to excessive cell death. Other studies have shown that 10 μM or above of haemin inhibit planktonic growth when pre-cultured from a rich iron-replete media (Torres et al., 2007). In this study, to mimic the conditions bacteria are exposed to during an infection, cells were pre-cultured in iron restricted media, and hemoglobin was shown not to be toxic to the biofilm in the time period studied. This finding highlights that tolerance to haemin toxicity depends on the iron status of the cells, and that iron starved cells, as would be likely in the initial stages of an infection, have a higher tolerance to haem toxicity.

The cell clusters found in biofilms grown with hemoglobin as main iron source could be a result of hemoglobin deposition on outer layers of the biofilm enhancing aerobic respiration in cells by providing haem as cofactor for the respiration enzymes, increasing their energy efficiency, and enabling faster growth in comparison to an overall anaerobic or hypoxic biofilm (Stewart and Franklin, 2008; Hammer and Skaar, 2011). Assessment of anaerobic marker *ldh1* in iron-treated biofilms (Richardson et al., 2008), showed a correlation to this hypothesis, downregulation of this *ldh1* with hemoglobin (Supplementary Figure S3). Excess, but not toxic, hemoglobin (or haem), can provide sufficient haem for the cells not needing their costly *de novo* haem biosynthetic pathway and leading to faster growth. Clusters could potentially give rise to tower-like structures over time with the constant stimulus of flow and hemoglobin, like that observed in catheter related infections (Raad, 1998).

We do show that Fur is regulating or positively interacting in other metabolic pathways responding to stresses which lead to cell death in the biofilm, since *Δfur* had considerably fewer dead cells in the presence of inorganic iron. Fifty percent of dead cells in biofilms of the iron restriction condition suggest that iron restriction in the body may be a signal inducing autolysis in the biofilm, leading to release of eDNA contributing to the matrix at least in *S. aureus* Newman. Studies have shown that expression of autolysin Atl and peptidoglycan hydrolase LytM is induced in low iron conditions in *S. aureus* SH1000 *Δfur* (Johnson et al., 2011). Overall, our results suggest that Fur has an iron-dependent role at initial adhesion of biofilms in matrix production and at later stages of biofilm maturation in cell death. Conversely, hemoglobin exposure in the biofilm did not cause changes in dead cell biomass in *Δfur* biofilms, confirming that Fur regulation of cell death is completely independent of hemoglobin.

The investigation of the expression of selected biofilm genes here has provided useful hints about how the biofilm senses the iron sources; however, RT-qPCR is an approach that requires substantial quality control to demonstrate meaningful results (Huggett et al., 2005; Fleige and Pfaffl, 2006). Despite the known instability of RNA, and RNA extraction from biofilms presenting extra difficulties, RNA from the biofilms in this study showed a good purity and integrity under the protocols described.

The gene expression analysis indicated that *S. aureus* biofilms are iron sufficient when provided with the high levels of iron (inorganic or hemoglobin) as demonstrated by ferritin gene expression results in the biofilms, responding as expected. HrtA expression allowed us to confirm that hemoglobin is specifically sensed by the biofilm cells. It has been shown Fur is repressed by iron in planktonic growth (Johnson et al., 2011); however, Fur showed an opposite trend in the biofilm, supporting different metabolic states and regulatory networks in both growth modes. Iron responsive expression of *isdB* varied between *S. aureus* Newman and the clinical isolates. The lack of response to iron seen in *S. aureus* Newman biofilms might be due to the presence of a unique mutation in Sae, causing constitutive or high expression of regulated genes like *isdB* (Mainiero et al., 2010; Johnson et al., 2011). *Staphylococcus aureus* Newman planktonic expression of *isdB* was induced in iron restriction as expected, confirming that particularly in the biofilm other regulatory effects must be involved to neutralize the iron-responsive expression (Figure 4B). *Staphylococcus aureus* Newman’s specific genetic feature (mutation in *saeS*) might explain the particular iron responsive pattern of one gene, however, it highlights that using just one strain for gene expression studies might overemphasize gene expression findings (Adhikari and Novick, 2008).

Expression of biofilm dispersal markers (*agrB* and *RNAIII*) confirmed that hemoglobin also induces an opposite gene expression response in *S. aureus* Newman biofilms compared to an inorganic iron source. With a repressed Agr QS system, adhesins are kept upregulated and proteases are kept downregulated, which favors the biofilm formation (Novick and Geisinger, 2008). Thus, hemoglobin, repressing the Agr QS system by a still unknown mechanism, is also affecting a large network of biofilm regulators. It has been reported that Agr induces Sae in low iron conditions in *S. aureus* Newman, which potentially can be the case in the presence of hemoglobin, if haemin is directly routed to membrane. Thus, Sae controls the expression of Emp, an important protein in the matrix composition of *S. aureus* Newman biofilms (Johnson et al., 2008, 2011). However, conflicting evidence toward our results showed that haemin through an unknown mechanism represses SaeS activity, which in turn downregulates a specific set of targets such as haemolysins (Schmitt et al., 2012). On the other hand, Fur represses Sae in the presence of iron, but Sae is also repressed by iron in a Fur–independent way, whereas both scenarios can explain the biofilm response to FeCl_3_ seen in our assays (Johnson et al., 2011).

Each clinical isolate studied here presented a different iron responsive gene expression pattern of the Agr QS system, not explained by their Agr type, since Newman and EC12 have Agr type I and BC03 has Agr type II. The responses of clinical isolates did not correlate to the common phenotypic response of increased biofilm matrix in the presence of hemoglobin. These results suggest that iron availability and source type might be an important stimulus in the biofilm associated infection and very specific to the isolate and its dissemination process. The broad range and variable responses seen in this pathogen might be just the tip of the iceberg of the complex networking and overlapping activities of regulators. Clinical isolates from endocarditis and bacteraemia infections were not strong biofilm formers under iron restriction; however, they both induced biofilm formation in the presence of hemoglobin, suggesting a widespread biofilm response to the provision of hemoglobin, possibly through a conserved mechanism. The expression of marker genes changed in intensity among isolates, raising the question what fold changes in gene expression are needed to actually trigger a relevant and differential phenotypic response?

In this study, PSM of the two main classes showed dissimilar response in *S. aureus* Newman biofilms with iron treatments. Overall, *psmα* did not change considerably with either iron source; however, *psmβ* presented a gene expression pattern matching RNAIII expression i.e., induced with inorganic iron and repressed with hemoglobin. This result is in agreement with at least two other studies regarding PSM and biofilms (Queck et al., 2008; Kaito et al., 2013). The first one reported that PSMs are regulated by AgrA not RNAIII in *S. aureus* MW2 (CA-MRSA), therefore in our study, we expected that PSM expression would coincide with RNAIII which is under control of AgrA (Queck et al., 2008). The second study showed that both classes of PSM have relatively the same importance in structuring *S. aureus* USA300 (CA-MRSA) biofilms when deleted from the chromosome. *Staphylococcus aureus* USA300 lacks *psm-mec*, which has been shown to change the expression of the other PSMs (Kaito et al., 2013), thus in this context the MRSA strain behavior should be applicable to a MSSA strain, like *S. aureus* Newman. Moreover, reports show that each one of the seven conserved PSMs in *S. aureus* isolates have different roles in spreading on wet surfaces. PSMα3 and PSMγ granted the most effective properties on promoting the spreading of *S. aureus* SH1000 and the whole PSMα operon deletion caused a more severe defect in spreading compared to PMSβ in *S. aureus* Newman (Tsompanidou et al., 2013). Thus, the differential expression of *psmα* and *psmβ* in response to inorganic iron and hemoglobin in *S. aureus* Newman could be implying different regulation mechanisms. Localized gene expression assays with promoter-reporter fusions might provide better insights about Agr and PSM expression and correlation to biofilm architecture in response to hemoglobin.

Global transcript analyses of *in vitro* and infection-derived *S. aureus* biofilms, e.g., using RNA sequencing approaches, should offer a more complete picture in the future. At present the effects of antimicrobial interventions (Singh et al., 2019; Wu et al., 2019), responses to sublethal disinfectant challenges (Slany et al., 2017), the importance of iron homeostasis in MRSA viability (Abouelhassan et al., 2018) and the differential response to immune cells (Scherr et al., 2013) have been reported for *S. aureus* following transcript analysis of biofilms.

*Staphylococcus aureus* biofilm control strategies are primarily focused on preventing adhesion of cells in device-related infections, discovering new biofilm essential molecules to target with drugs or to develop vaccines. In this study, we show that biofilms can respond to the specific local conditions, and conclude that an understanding of this is essential to develop targeted prevention or treatment strategies. Future studies should consider the broad impact that media components may have on the results obtained, especially where the *in vivo* situation is being modeled. More specifically, the location of an infection (e.g., bloodstream, implanted devices, skin, and soft tissue) may influence bacterial responses differently depending upon the balance of iron sources available and should be considered in the future. In a real-life infection, a combination of iron sources will be available as hemoglobin and Fe^3+^ scavenged by siderophores from ferritin and transferrins. Skaar et al. (2004) have shown that for *S. aureus* Hb is preferentially used, and so in our *in vitro* model we speculate that the differential responses we see here could manifest as a phenotypic transition (biofilm to dispersal) in response to a change in the preferred iron source (Hb to Fe^3+^). The application of state-of-the-art transcriptomic, proteomic, and metabolomic approaches in the future may allow linkage of gene expression changes to phenotypic responses in localized areas of the biofilm. The mechanistic insights gained may be exploited to develop new ways to enhance the treatment of infections by the disruption of biofilms.

## Data Availability Statement

All datasets generated for this study are included in the article/Supplementary Material.

## Author Contributions

PD-S, SW, and SS contributed to experimental design and analysis of results. PD-S contributed to experimental work. PD-S and SS contributed to writing of the paper. All authors contributed to the article and approved the submitted version.

### Conflict of Interest

The authors declare that the research was conducted in the absence of any commercial or financial relationships that could be construed as a potential conflict of interest.

## Supplementary Material

Supplementary Figure 1 Newman ∆fur phenotypic changes. **(A)**, SDS-PAGE gel of cell wall proteins normally regulated by Fur. Growth in presence of iron is denoted by +Fe and in the absence of iron by –Fe. Lack of Fur is shown by similar cell wall protein composition in +Fe/–Fe. L is Protein ladder (High Range Rainbow Marker). Ct plots obtained for fur and gyrB RT-qPCRs in Newman ∆fur **(B)** and Newman WT **(C)**. Absence of fur RNA is shown by high Ct values, which represents unspecific amplification.

Supplementary Figure 2 | Newman biofilm viability assayed by resazurin. A *Staphylococcus aureus* culture grown in RPMI overnight resuspended in fresh medium was diluted to a final concentration of 1 x 10^7^ CFU ml^-1^ in 200 μl of desired medium (RPMI or RPMI supplemented with 2 and 40 µM of FeCl_3_ or 0.5 and 10 µM hemoglobin) in a sterile 96-well microtitre plate. The microplate was incubated at 37°C and 200 r.p.m for desired time. Spent medium was removed and biofilms were washed twice with PBS. Biofilm viability was assessed by resazurin.

Supplementary Figure 3 | *ldh1* expression in Newman biofilms. Data is plotted as mean of three biological replicates each including technical triplicates.

Supplementary Figure 4 **(A)** Agr typing in EC12 and BC03 clinical strains. PCR amplification of specific Agr types. 1, 1 kb DNA ladder (Zymo Research); 2, BC01; **3**, **BC03**; 4, BC07; 5, BC08; 6, EC04; 7, EC06; 8, EC08; **9**, **EC12**; 10, EC13; 11, negative control; 12, BC01; **13**, **BC03**; 14, BC07; 15, BC08; 16, EC04; 17, EC06; 18, EC08; **19**, **EC12**; 20, 1 kb DNA ladder (Zymo Research). Bold annotation represents strains included in this study.

Supplementary Table 1 Strains, plasmids and primers used in this study.

## References

[ref1] Abouelhassan Y. Zhang Y. Jin S. Huigens R. W. (2018). Transcript profiling of MRSA biofilms treated with a halogenated phenazine eradicating agent: a platform for defining cellular targets and pathways critical to biofilm survival. Angew. Chem. Int. Ed. Eng. 57, 15523–15528. 10.1002/anie.201809785, PMID: 30230671 PMC6407420

[ref2] Adhikari R. P. Novick R. P. (2008). Regulatory organization of the staphylococcal sae locus. Microbiology 154, 949–959. 10.1099/mic.0.2007/012245-0, PMID: 18310041

[ref3] Atshan S. S. Shamsudin M. N. Karunanidhi A. van Belkumd A. Lunga L. T. T. Sekawi Z.. (2013). Quantitative PCR analysis of genes expressed during biofilm development of methicillin resistant *Staphylococcus aureus* (MRSA). Infect. Genet. Evol. 18, 106–112. 10.1016/j.meegid.2013.05.002, PMID: 23669446

[ref4] Bae T. Schneewind O. (2006). Allelic replacement in *Staphylococcus aureus* with inducible counter-selection. Plasmid 55, 58–63. 10.1016/j.plasmid.2005.05.005, PMID: 16051359

[ref5] Beenken K. Blevins J. Smeltzer M. (2003). Mutation of sarA in *Staphylococcus aureus* limits biofilm formation. Infect. Immun. 71, 4206–4211. 10.1128/IAI.71.7.4206-4211.2003, PMID: 12819120 PMC161964

[ref6] Boles B. R. Horswill A. R. (2008). Agr-mediated dispersal of *Staphylococcus aureus* biofilms. PLoS Pathog. 4:e1000052. 10.1371/journal.ppat.1000052, PMID: 18437240 PMC2329812

[ref7] Boles B. R. Thoendel M. Roth A. J. Horswill A. R. (2010). Identification of genes involved in polysaccharide-independent *Staphylococcus aureus* biofilm formation. PLoS One 5:e10146. 10.1371/journal.pone.0010146, PMID: 20418950 PMC2854687

[ref8] Carver P. (2018). The battle for iron between humans and microbes. Curr. Med. Chem. 25, 85–96. 10.2174/0929867324666170720110049, PMID: 28730969

[ref9] Casabona M. G. Kneuper H. Alferes de Lima D. Harkins C. P. Zoltner M. Hjerde E.. (2017). Haem-iron plays a key role in the regulation of the Ess/type VII secretion system of *Staphylococcus aureus* RN6390. Microbiology 163, 1839–1850. 10.1099/mic.0.000579, PMID: 29171824 PMC5845736

[ref10] Costerton J. W. Stewart P. S. Greenberg E. P. (1999). Bacterial biofilms: a common cause of persistent infections. Science 284, 1318–1322. 10.1126/science.284.5418.1318, PMID: 10334980

[ref11] Crichton R. (2001). Inorganic biochemistry of iron metabolism: From molecular mechanisms to clinical consequences. 2nd Edn. Chichester, England: John Wiley & Sons.

[ref12] Dauros-Singorenko P. Swift S. (2014). The transition from iron starvation to iron sufficiency as an important step in the progression of infection. Sci. Prog. 97, 371–382. 10.3184/003685014X14151846374739, PMID: 25638949 PMC10365408

[ref13] Duthie E. S. Lorenz L. L. (1952). Staphylococcal coagulase: mode of action and antigenicity. J. Gen. Microbiol. 6, 95–107. 10.1099/00221287-6-1-2-95, PMID: 14927856

[ref14] Escolar L. Perez-Martin J. Lorenzo V. (1999). Opening the iron box: transcriptional metalloregulation by the fur protein. J. Bacteriol. 181, 6223–6229. 10.1128/JB.181.20.6223-6229.1999, PMID: 10515908 PMC103753

[ref15] Fleige S. Pfaffl M. W. (2006). RNA integrity and the effect on the real-time qRT-PCR performance. Mol. Aspects Med. 27, 126–139. 10.1016/j.mam.2005.12.003, PMID: 16469371

[ref16] Flemming H. C. Wingender J. (2010). The biofilm matrix. Nat. Rev. Microbiol. 8, 623–633. 10.1038/nrmicro2415, PMID: 20676145

[ref17] Fux C. Shirtliff M. Stoodley P. Costerton J. (2005). Can laboratory reference strains mirror "real-world" pathogenesis? Trends Microbiol. 13, 58–63. 10.1016/j.tim.2004.11.001, PMID: 15680764

[ref18] Günther F. Wabnitz G. Stroh P. Prior B. Obst U. Samstag Y.. (2009). Host defence against *Staphylococcus aureus* biofilms infection: phagocytosis of biofilms by polymorphonuclear neutrophils (PMN). Mol. Immunol. 46, 1805–1813. 10.1016/j.molimm.2009.01.020, PMID: 19261332

[ref19] Haley K. P. Skaar E. P. (2012). A battle for iron: host sequestration and *Staphylococcus aureus* acquisition. Microbes Infect. 14, 217–227. 10.1016/j.micinf.2011.11.001, PMID: 22123296 PMC3785375

[ref20] Hammer N. D. Skaar E. P. (2011). Molecular mechanisms of *Staphylococcus aureus* iron acquisition. Annu. Rev. Microbiol. 65, 129–147. 10.1146/annurev-micro-090110-102851 21639791 PMC3807827

[ref21] Hellemans J. Mortier G. De Paepe A. Speleman F. Vandesompele J. (2007). qBase relative quantification framework and software for management and automated analysis of real-time quantitative PCR data. Genome Biol. 8:R19. 10.1186/gb-2007-8-2-r19, PMID: 17291332 PMC1852402

[ref22] Heydorn A. Nielsen A. T. Hentzer M. Sternberg C. Givskov M. Ersbøll B. K.. (2000). Quantification of biofilm structures by the novel computer program COMSTAT. Microbiology 146, 2395–2407. 10.1099/00221287-146-10-2395, PMID: 11021916

[ref23] Huggett J. Dheda K. Bustin S. Zumla A. (2005). Real-time RT-PCR normalisation; strategies and considerations. Genes Immun. 6, 279–284. 10.1038/sj.gene.6364190, PMID: 15815687

[ref24] Hurd A. F. Garcia-Lara J. Rauter Y. Cartron M. Foster S. J. (2012). The iron-regulated surface proteins IsdA, IsdB, and IsdH are not required for heme iron utilization in *Staphylococcus aureus*. FEMS Microbiol. Lett. 329, 93–100. 10.1111/j.1574-6968.2012.02502.x, PMID: 22268825

[ref25] Jenul C. Horswill A. R. (2018). Regulation of *Staphylococcus aureus* virulence. Microbiol. Spectr. 7. 10.1128/microbiolspec.GPP3-0031-2018, PMID: 30953424 PMC6452892

[ref26] Johnson M. Cockayne A. Morrissey J. A. (2008). Iron-regulated biofilm formation in *Staphylococcus aureus* Newman requires *ica* and the secreted protein Emp. Infect. Immun. 76, 1756–1765. 10.1128/IAI.01635-07, PMID: 18268030 PMC2292859

[ref27] Johnson M. Cockayne A. Williams P. H. Morrissey J. A. (2005). Iron-responsive regulation of biofilm formation in *Staphylococcus aureus* involves fur-dependent and fur-independent mechanisms. J. Bacteriol. 187, 8211–8215. 10.1128/JB.187.23.8211-8215.2005, PMID: 16291697 PMC1291266

[ref28] Johnson M. Sengupta M. Purves J. Tarrant E. Williams P. H. Cockayne A.. (2011). Fur is required for the activation of virulence gene expression through the induction of the sae regulatory system in *Staphylococcus aureus*. Int. J. Med. Microbiol. 301, 44–52. 10.1016/j.ijmm.2010.05.003, PMID: 20705504 PMC2994983

[ref29] Kaito C. Saito Y. Ikuo M. Omae Y. Mao H. Nagano G.. (2013). Mobile genetic element SCCmec-encoded psm-mec RNA suppresses translation of agrA and attenuates MRSA virulence. PLoS Pathog. 9:e1003269. 10.1371/journal.ppat.1003269, PMID: 23592990 PMC3617227

[ref30] Kim H. K. DeDent A. Cheng A. G. McAdow M. Bagnoli F. Missiakas D. M.. (2010). IsdA and IsdB antibodies protect mice against *Staphylococcus aureus* abscess formation and lethal challenge. Vaccine 28, 6382–6392. 10.1016/j.vaccine.2010.02.097, PMID: 20226248 PMC3095377

[ref31] Kraemer G. Iandolo J. J. (1990). High-frequency transformation of *Staphylococcus aureus* by electroporation. Curr. Microbiol. 21, 373–376. 10.1007/BF02199440

[ref32] Kreiswirth B. Lofdahl S. Betley M. O’Reilly M. Schlievert P. Bergdoll M.. (1983). The toxic shock syndrome exotoxin structural gene is not detectably transmitted by a prophage. Nature 305, 709–712. 10.1038/305709a0, PMID: 6226876

[ref33] Laakso H. A. Marolda C. L. Pinter T. B. Stillman M. J. Heinrichs D. E. (2016). A heme-responsive regulator controls synthesis of staphyloferrin B in *Staphylococcus aureus*. J. Biol. Chem. 291, 29–40. 10.1074/jbc.M115.696625, PMID: 26534960 PMC4697164

[ref34] Leid J. G. Shirtliff M. E. Costerton J. W. Stoodley P. (2002). Human leukocytes adhere to, penetrate, and respond to *Staphylococcus aureus* biofilms. Infect. Immun. 70, 6339–6345. 10.1128/IAI.70.11.6339-6345.2002, PMID: 12379713 PMC130380

[ref35] Lister J. L. Horswill A. R. (2014). *Staphylococcus aureus* biofilms: recent developments in biofilm dispersal. Front. Cell. Infect. Microbiol. 4:178. 10.3389/fcimb.2014.00178, PMID: 25566513 PMC4275032

[ref36] Mainiero M. Goerke C. Geiger T. Gonser C. Herbert S. Wolz C. (2010). Differential target gene activation by the *Staphylococcus aureus* two-component system *saeRS*. J. Bacteriol. 192, 613–623. 10.1128/JB.01242-09, PMID: 19933357 PMC2812464

[ref37] Mann E. E. Rice K. C. Boles B. R. Endres J. L. Ranjit D. Chandramohan L.. (2009). Modulation of eDNA release and degradation affects *Staphylococcus aureus* biofilm maturation. PLoS One 4:e5822. 10.1371/journal.pone.0005822, PMID: 19513119 PMC2688759

[ref38] Mazmanian S. K. Skaar E. P. Gaspar A. H. Humayun M. Gornicki P. Jelenska J.. (2003). Passage of heme-iron across the envelope of *Staphylococcus aureus*. Science 299, 906–909. 10.1126/science.1081147, PMID: 12574635

[ref39] Miajlovic H. Zapotoczna M. Geoghegan J. A. Kerrigan S. W. Speziale P. Foster T. J. (2010). Direct interaction of iron-regulated surface determinant IsdB of *Staphylococcus aureus* with the GPIIb/IIIa receptor on platelets. Microbiology 156, 920–928. 10.1099/mic.0.036673-0, PMID: 20007649

[ref40] Morrissey J. A. Cockayne A. Brummell K. Williams P. (2004). The staphylococcal ferritins are differentially regulated in response to iron and manganese and via PerR and Fur. Infect. Immun. 72, 972–979. 10.1128/iai.72.2.972-979.2004, PMID: 14742543 PMC321569

[ref41] Novick R. P. (1991). Genetic systems in staphylococci. Methods Enzymol. 204, 587–636. 10.1016/0076-6879(91)04029-n, PMID: 1658572

[ref42] Novick R. P. Geisinger E. (2008). Quorum sensing in staphylococci. Annu. Rev. Genet. 42, 541–564. 10.1146/annurev.genet.42.110807.091640, PMID: 18713030

[ref44] Olsen J. (2015). Biofilm-specific antibiotic tolerance and resistance. Eur. J. Clin. Microbiol. Infect. Dis. 34, 877–886. 10.1007/s10096-015-2323-z, PMID: 25630538

[ref45] Oogai Y. Matsuo M. Hashimoto M. Kato F. Sugai M. Komatsuzawa H. (2011). Expression of virulence factors by *Staphylococcus aureus* grown in serum. Appl. Environ. Microbiol. 77, 8097–8105. 10.1128/AEM.05316-11, PMID: 21926198 PMC3208999

[ref43] O’Toole G. Kaplan H. B. Kolter R. (2000). Biofilm formation as microbial development. Annu. Rev. Microbiol. 54, 49–79. 10.1146/annurev.micro.54.1.49, PMID: 11018124

[ref46] Otto M. (2013). Staphylococcal infections: mechanisms of biofilm maturation and detachment as critical determinants of pathogenicity. Annu. Rev. Med. 64, 175–188. 10.1146/annurev-med-042711-140023, PMID: 22906361

[ref47] Otto M. (2018). Staphylococcal biofilms. Microbiol. Spectr. 6. 10.1128/microbiolspec.GPP3-0023-2018, PMID: 30117414 PMC6282163

[ref48] Payne D. E. Boles B. R. (2015). Emerging interactions between matrix components during biofilm development. Curr. Genet. 62, 137–141. 10.1007/s00294-015-0527-5, PMID: 26515441 PMC4723619

[ref49] Periasamy S. Joo H. S. Duong A. C. Bach T. H. L. Tan V. Y. Chatterjee S. S.. (2012). How *Staphylococcus aureus* biofilms develop their characteristic structure. Proc. Natl. Acad. Sci. U. S. A. 109, 1281–1286. 10.1073/pnas.1115006109, PMID: 22232686 PMC3268330

[ref50] Peschel A. Otto M. (2013). Phenol-soluble modulins and staphylococcal infection. Nat. Rev. Microbiol. 11, 667–673. 10.1038/nrmicro3110, PMID: 24018382 PMC4780437

[ref51] Pishchany G. Sheldon J. R. Dickson C. F. Alam M. D. T. Read T. D. Gell D. A.. (2014). IsdB-dependent hemoglobin binding is required for acquisition of heme by *Staphylococcus aureus*. J. Infect. Dis. 209, 1764–1772. 10.1093/infdis/jit817, PMID: 24338348 PMC4038968

[ref52] Pragman A. Schlievert P. (2004). Virulence regulation in *Staphylococcus aureus*: the need for in vivo analysis of virulence factor regulation. FEMS Immunol. Med. Microbiol. 42, 147–154. 10.1016/j.femsim.2004.05.005, PMID: 15364098

[ref53] Pratten J. Foster S. Chan P. Wilson M. Nair S. (2001). *Staphylococcus aureus* accessory regulators: expression within biofilms and effect on adhesion. Microbes Infect. 3, 633–637. 10.1016/s1286-4579(01)01418-6, PMID: 11445449

[ref54] Pynnonen M. Stephenson R. E. Schwartz K. Hernandez M. Boles B. R. (2011). Hemoglobin promotes *Staphylococcus aureus* nasal colonization. PLoS Pathog. 7:e1002104. 10.1371/journal.ppat.1002104, PMID: 21750673 PMC3131264

[ref55] Queck S. Y. Jameson-Lee M. Villaruz A. E. Bach T. L. Burhan A. Sturdevant D. E.. (2008). RNAIII-independent target gene control by the agr quorum-sensing system: insight into the evolution of virulence regulation in *Staphylococcus aureus*. Mol. Cell 32, 150–158. 10.1016/j.molcel.2008.08.005, PMID: 18851841 PMC2575650

[ref56] Raad I. (1998). Intravascular-catheter-related infections. Lancet 351, 893–898. 10.1016/S0140-6736(97)10006-X 9525387

[ref57] Ramakers C. Ruijter J. M. Deprez R. H. L. Moorman A. F. M. (2003). Assumption-free analysis of quantitative real-time polymerase chain reaction (PCR) data. Neurosci. Lett. 339, 62–66. 10.1016/S0304-3940(02)01423-4, PMID: 12618301

[ref58] Ratledge C. Dover L. G. (2000). Iron metabolism in pathogenic bacteria. Annu. Rev. Microbiol. 54, 881–941. 10.1146/annurev.micro.54.1.881, PMID: 11018148

[ref59] Reniere M. Torres V. J. Skaar E. P. (2007). Intracellular metalloporphyrin metabolism in *Staphylococcus aureus*. Biometals 20, 333–345. 10.1007/s10534-006-9032-0, PMID: 17387580

[ref60] Richardson A. R. Libby S. J. Fang F. C. (2008). A nitric oxide-inducible lactate dehydrogenase enables *Staphylococcus aureus* to resist innate immunity. Science 319, 1672–1676. 10.1126/science.1155207, PMID: 18356528

[ref61] Schenk S. Laddaga R. A. (1992). Improved method for electroporation of *Staphylococcus aureus*. FEMS Microbiol. Lett. 94, 133–138. 10.1111/j.1574-6968.1992.tb05302.x, PMID: 1521761

[ref62] Scherr T. D. Roux C. M. Hanke M. L. Angle A. Dunman P. M. Kieliana T. (2013). Global transcriptome analysis of *Staphylococcus aureus* biofilms in response to innate immune cells. Infect. Immun. 81, 4363–4376. 10.1128/IAI.00819-13, PMID: 24042108 PMC3837966

[ref63] Schlievert P. Case L. Nemeth K. Davis K. Sun Y. Qin W.. (2007). Alpha and beta chains of hemoglobin inhibit production of *Staphylococcus aureus* exotoxins. Biochemistry 46, 14349–14358. 10.1021/bi701202w, PMID: 18020451 PMC2435367

[ref64] Schmitt J. Joost I. Skaar E. P. Herrmann M. Bischoff M. (2012). Haemin represses the haemolytic activity of *Staphylococcus aureus* in a Sae-dependent manner. Microbiology 158, 2619–2631. 10.1099/mic.0.060129-0, PMID: 22859613 PMC4083625

[ref65] Schröder I. Johnson E. de Vries S. (2003). Microbial ferric iron reductases. FEMS Microbiol. Rev. 27, 427–447. 10.1016/S0168-6445(03)00043-3, PMID: 12829278

[ref66] Sepúlveda I. Salazar J. C. García-Angulo V. A. (2018). Overview on the bacterial iron-riboflavin metabolic axis. Front. Microbiol. 9:1478. 10.3389/fmicb.2018.01478, PMID: 30026736 PMC6041382

[ref67] Singh N. Rajwade J. Paknikara K. M. (2019). Transcriptome analysis of silver nanoparticles treated *Staphylococcus aureus* reveals potential targets for biofilm inhibition. Colloids Surf. B: Biointerfaces 175, 487–497. 10.1016/j.colsurfb.2018.12.032, PMID: 30572157

[ref68] Skaar E. P. Humayun M. Bae T. DeBord K. L. Schneewind O. (2004). Iron-source preference of *Staphylococcus aureus* infections. Science 305, 1626–1628. 10.1126/science.1099930, PMID: 15361626

[ref69] Slany M. Oppelt J. Cincarova L. (2017). Formation of *Staphylococcus aureus* biofilm in the presence of sublethal concentrations of disinfectants studied via a transcriptomic analysis using transcriptome sequencing (RNA-seq). Appl. Environ. Microbiol. 83, e01643–e01717. 10.1128/AEM.01643-17, PMID: 29030437 PMC5717214

[ref70] Stewart P. S. Franklin M. J. (2008). Physiological heterogeneity in biofilms. Nat. Rev. Microbiol. 6, 199–210. 10.1038/nrmicro1838, PMID: 18264116

[ref71] Strommenger B. Cuny C. Werner G. Witte W. (2004). Obvious lack of association between dynamics of epidemic methicillin-resistant *Staphylococcus aureus* in Central Europe and agr specificity groups. Eur. J. Clin. Microbiol. Infect. Dis. 23, 15–19. 10.1007/s10096-003-1046-8, PMID: 14652782

[ref72] Torres V. J. Pishchany G. Humayun M. Schneewind O. Skaar E. P. (2006). *Staphylococcus aureus* IsdB is a hemoglobin receptor required for heme iron utilization. J. Bacteriol. 188, 8421–8429. 10.1128/JB.01335-06, PMID: 17041042 PMC1698231

[ref73] Torres V. J. Stauff D. L. Pishchany G. Bezbradica J. S. Gordy L. E. Iturregui J.. (2007). A *Staphylococcus aureus* regulatory system that responds to host heme and modulates virulence. Cell Host Microbe 1, 109–119. 10.1016/j.chom.2007.03.001, PMID: 18005689 PMC2083280

[ref74] Toté K. Vanden Berghe D. Maes L. Cos P. (2008). A new colorimetric microtitre model for the detection of *Staphylococcus aureus* biofilms. Lett. Appl. Microbiol. 46, 249–254. 10.1111/j.1472-765X.2007.02298.x, PMID: 18069978

[ref75] Troxell B. Hassan H. M. (2013). Transcriptional regulation by ferric uptake regulator (Fur) in pathogenic bacteria. Front. Cell. Infect. Microbiol. 3:59. 10.3389/fcimb.2013.00059, PMID: 24106689 PMC3788343

[ref76] Tsompanidou E. Denham E. L. Becher D. de Jong A. Buist G. van Oosten M.. (2013). Distinct roles of phenol-soluble modulins in spreading of *Staphylococcus aureus* on wet surfaces. Appl. Environ. Microbiol. 79, 886–895. 10.1128/AEM.03157-12, PMID: 23183971 PMC3568538

[ref77] Valihrach L. Demnerova K. (2012). Impact of normalization method on experimental outcome using RT-qPCR in *Staphylococcus aureus*. J. Microbiol. Methods 90, 214–216. 10.1016/j.mimet.2012.05.008, PMID: 22613804

[ref78] Wu S. Liu Y. Zhang H. Lei L. (2019). The pathogenicity and transcriptome analysis of methicillin-resistant *Staphylococcus aureus* in response to water extract of *Galla chinensis*. Evid. Based Complementary Altern. Med. 2019:3276156. 10.1155/2019/3276156, PMID: 31379958 PMC6662456

[ref79] Zandri G. Pasquaroli S. Vignaroli C. Talevi S. Manso E. Donelli G.. (2012). Detection of viable but non-culturable staphylococci in biofilms from central venous catheters negative on standard microbiological assays. Clin. Microbiol. Infect. 18, E259–E261. 10.1111/j.1469-0691.2012.03893.x, PMID: 22578149

[ref80] Zapotoczna M. Jevnikar Z. Miajlovic H. Kos J. Foster T. J. (2013). Iron-regulated surface determinant B (IsdB) promotes *Staphylococcus aureus* adherence to and internalization by non-phagocytic human cells. Cell. Microbiol. 15, 1026–1041. 10.1111/cmi.12097, PMID: 23279065

