# Supplementary figures and images for: Staphylococcus aureus Biofilms and Their Response to a Relevant in vivo Iron Source
Source: Front Microbiol. 2020 Dec 21;11:509525. doi: 10.3389/fmicb.2020.509525 (PMC7779473; doi:10.3389/fmicb.2020.509525)

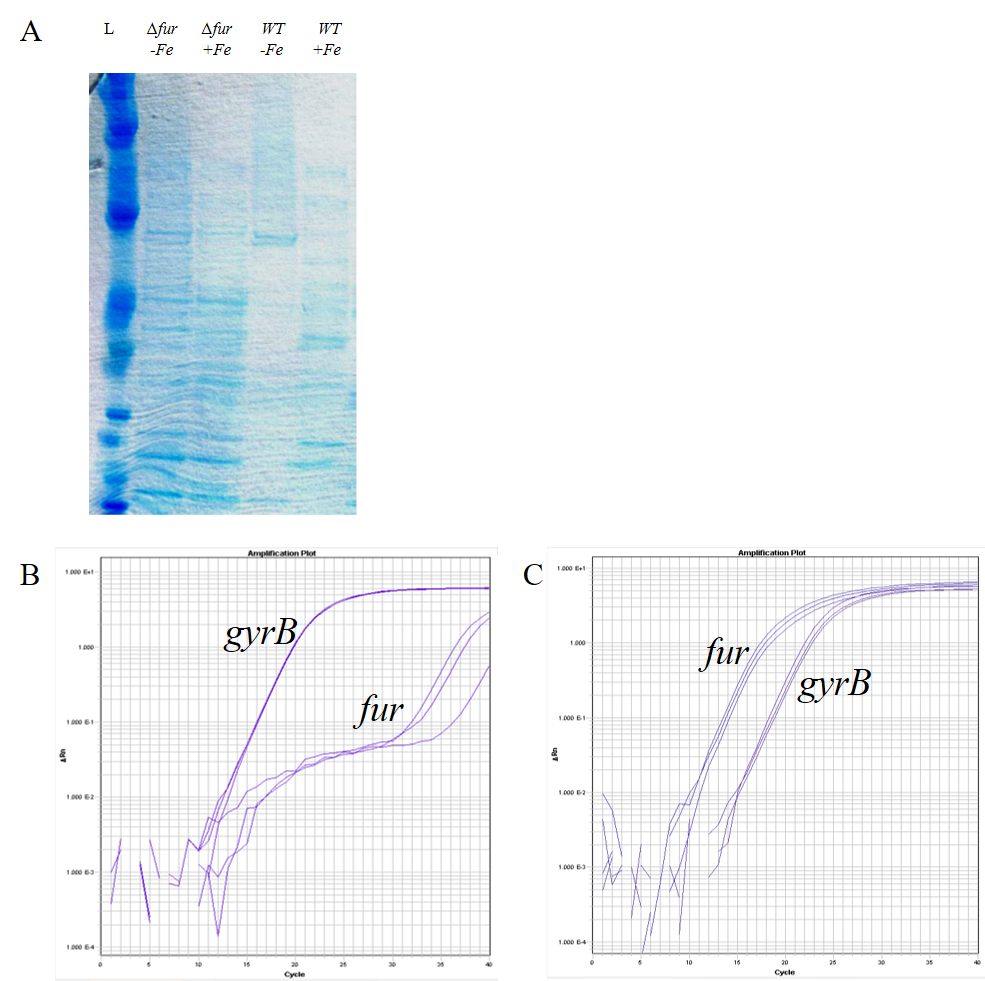

Supplement: Supplementary Figure 1 — Newman ∆fur phenotypic changes. (A), SDS-PAGE gel of cell wall proteins normally regulated by Fur. Growth in presence of iron is denoted by +Fe and in the absence of iron by –Fe. Lack of Fur is shown by similar cell wall protein composition in +Fe/–Fe. L is Protein ladder (High Range Rainbow Marker). Ct plots obtained for fur and gyrB RT-qPCRs in Newman ∆fur (B) and Newman WT (C). Absence of fur RNA is shown by high Ct values, which represents unspecific amplification. [file Image_1.tif]

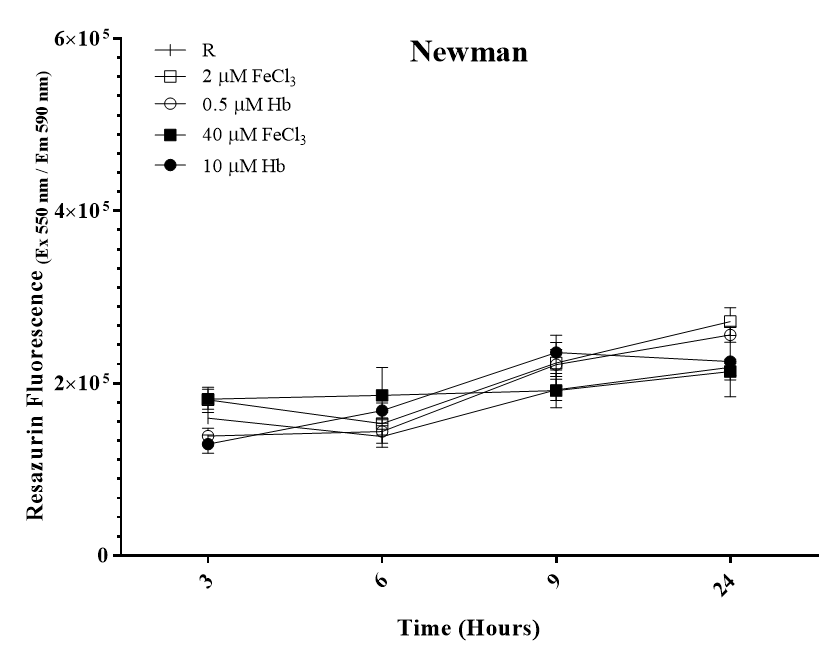

Supplement: Supplementary Figure 2 — | Newman biofilm viability assayed by resazurin. A Staphylococcus aureus culture grown in RPMI overnight resuspended in fresh medium was diluted to a final concentration of 1 x 10^7^ CFU ml-1 in 200 μl of desired medium (RPMI or RPMI supplemented with 2 and 40 µM of FeCl3 or 0.5 and 10 µM hemoglobin) in a sterile 96-well microtitre plate. The microplate was incubated at 37°C and 200 r.p.m for desired time. Spent medium was removed and biofilms were washed twice with PBS. Biofilm viability was assessed by resazurin. [file Image_2.tif]

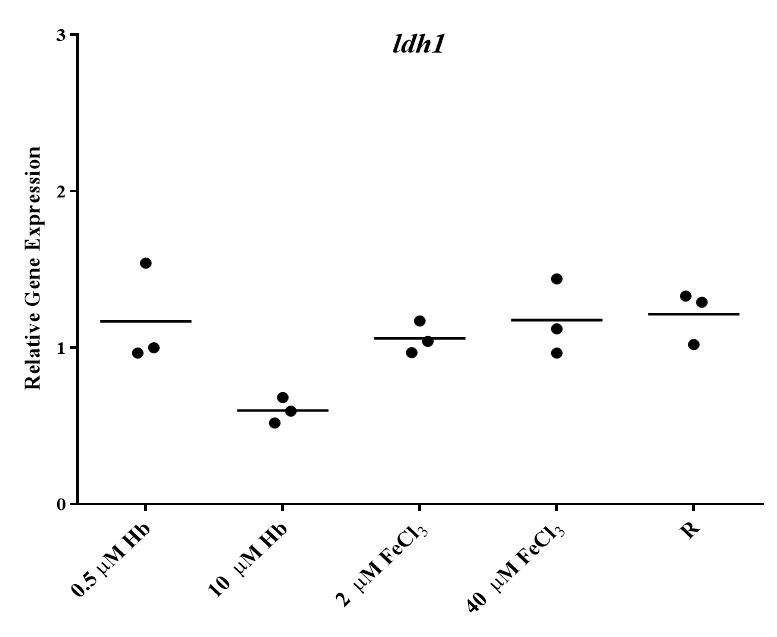

Supplement: Supplementary Figure 3 [file Image_3.tif]

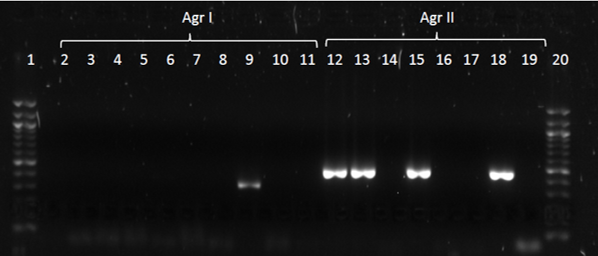

Supplement: Supplementary Figure 4 — (A) Agr typing in EC12 and BC03 clinical strains. PCR amplification of specific Agr types. 1, 1 kb DNA ladder (Zymo Research); 2, BC01; 3, BC03; 4, BC07; 5, BC08; 6, EC04; 7, EC06; 8, EC08; 9, EC12; 10, EC13; 11, negative control; 12, BC01; 13, BC03; 14, BC07; 15, BC08; 16, EC04; 17, EC06; 18, EC08; 19, EC12; 20, 1 kb DNA ladder (Zymo Research). Bold annotation represents strains included in this study. [file Image_4.tif]
