# Supplementary material for: Staphylococcus aureus Biofilms and Their Response to a Relevant in vivo Iron Source
Source: Front Microbiol. 2020 Dec 21;11:509525. doi: 10.3389/fmicb.2020.509525 (PMC7779473; doi:10.3389/fmicb.2020.509525)
Supplement: Supplementary Table 1 [file Table_1.docx]

Table S1. Strains, plasmids and primers used in this study.

*Att* sites are shown in bold sequences and restriction sites are shown in underlined-italicized sequences.

| **Strains** | **Genotype or relevant features** |  |  | **Reference** |
| --- | --- | --- | --- | --- |
| Newman | *S. aureus,* 25904 ATCC |  |  | Duthie and Lorenz, 1952 |
| Newman *∆fur* | *S. aureus,* Newman *fur* deletion mutant |  |  | This study |
| BC03 | *S. aureus,* clinical isolate from a bacteraemia infection |  |  | This study |
| EC12 | *S. aureus,* clinical isolate endocarditis infection |  |  | This study |
| RN4220 | *S. aureus,* Restriction deficient strain  (*sau1,* *hsdR*), derived from NCTC 8325-4, ATCC 35556 |  |  | Kreiswirth et al., 1983 |
| TOP10 | *Escherichia coli,* chemically competent cells  F-, *mcrA* Δ( *mrr-hsdRMS-mcrBC*), Φ80*lacZ*ΔM15, Δ*lacX74*, *recA*, *araD*, Δ(*ara, leu*)7697, *galU*, *galK*, *rpsL*, *endA*, *nupG* |  |  | Thermo Fisher Scientific |
| DB3.1 | *Escherichia coli,* *gyrA*, *endA*, ∆(*sr1-recA*), *mcrB*, *mrr*, *hsdS*, *glnV44*, *ara*, *galK*, *lacY*, *proA*, *rpsL*, *xyl*, *leuB*, *mtl* |  |  | Thermo Fisher Scientific |
| **Plasmids** | **Genotype or relevant features** |  | **Vector size (bp)** |  |
| pKOR1 | Gateway Destination vector, attP1 and attP2 sites, PaTc::secY, AmpR(G-), CmR(G+) |  | 10030 | Bae and Schneewind, 2006 |
| pFK | fur-1-kb-upstream :: fur- 1-kb-downstream, in pKOR1, Cm^R^ |  | 9800 | This study |
| **PCR Primers** | **Sequence 5’-3’** | **Target DNA** | **Amplicon size (bp)** |  |
| Fur-up-F-attB2 | **GGGGACCACTTTGTACAAGAAAGCTGGGT**ACAACAACGCTTTTCAGATTTAG | 1 kb upstream region of *fur* |  | This study |
| Fur-up-R-sacII | AGGTTC*CCGCGG*TATTTCAACACTCATCTATATCACC | 1 kb upstream region of *fur* | 1046 | This study |
| Fur-down-F-sacII | GTTTCA*CCGCGG*AATAATTTAACTTTGGTAGTATGA | 1 kb downstream region of *fur* |  | This study |
| Fur-down-R-attB1 | **GGGGACSSGTTTGTACAAAAAGCAGGC**ACTAGCTAATGCAGCGCGGATC | 1 kb downstream region of *fur* | 1034 | This study |
| **RT-qPCR Primers** | Sequence 5’-3’ | Target DNA | Amplicon size (bp) |  |
| RNAIII-F  RNAIII-R | GTTTCACTGTGTCGATAATCCATTTT  AAGGAGTGATTTCAATGGCACAA | *RNAIII* | 77 | This study |
| AgrB-F  AgrB-R | GCCCATTCCTGTGCGACTTAT  GGCTCTTTGATGATAAGTGTGATAATG | *agrB* | 93 | This study |
| AgrII-F  AgrII-R | TACAAGTTCAAACGGTGATAG  CGACATTATAAGTATTACAACAGAG | *agr II* | 91 | This study |
| Fur-F  Fur-R | TGGAAAATCAGGTGAAAGGTGCTT  TGCGACAAGCAGGCGTAA | *Fur* | 119 | This study |
| IsdB-F  IsdB-R | CGCTTCTCTAAGTTCCTGATTCAAA  TGAGGCCCCTACTTCTGAAACA | *isdB* | 139 | This study |
| HrtA-F  HrtA-R | CTTTTGTTGCTCTATCGGCATCTAA  GCTTGAATGTATATCCGCATCAGT | *hrtA* | 138 | This study |
| Ftn-F  Ftn-R | AATCGCTCGTCAAGATAAAGATTATG  CGCCGATACGAGTTAAATAATTGA | *ftn* | 119 | This study |
| Psmα-F  Psmα-R | TAGTACCTACAATAGCCATC  GGTAACAACTAATCTCAAAC | *psm*α | 94 | This study |
| Psmβ-F  Psmβ-R | GGCGTAGGTTTATTAGGTAAATTAT  AAATCTCTCCCTCACCTTTG | *psmβ* | 90 | This study |
| Ldh1-F  Ldh1-R | AAAACATGCCACACCATATTCTCC  TACTAAATCTAAACGTGTTTCTCC | *ldh-1* | 136 | Richardson et al., 2008 |
| 16S-F  16S-R | AATACAAAGGGCAGCGAAACC  TCCGAACTGAGAACAACTTTATGG | *16S* | 62 | This study |
| GyrB-F  GyrB-R | CGATTGCTCTAGTAAAAGTCCTGAAG  TAGACCCCCCGGCAGAGT | *gyrB* | 71 | This study |
| Pta-F  Pta-R | AAAGCGCCAGGTGCTAAATTAC  CTGGACCAACTGCATCATATCC | *pta* | 121 | Valihrach and Demnerova, 2012 |
| Tpi-F  Tpi-R | TTCTGAACGTCGTGAATTATTCC  TTCACGCTCTTCGTCTGTTTC | *tpi* | 121 | Valihrach and Demnerova, 2012 |
| Hu-F  Hu-R | TTTACGTGCAGCACGTTCAC  AAAAAGAAGCTGGTTCAGCAGTAG | *hu* | 125 | Valihrach and Demnerova, 2012 |
| agrI-F  agrI-R | CACTTATCATCAAAGAGC  CCACTAATTATAGCTGG | Agr I |  | Strommenger et al., 2004 |
| agrII-F  agrII-R | GTAGAGCCGTATTGATTC  GTATTTCATCTCTTTAAGG | Agr II |  | Strommenger et al., 2004 |
| agrIII-F  agrIII-R | CAAGCTATTACATTACTACCA  AATGCTTCCACTTACTATC | Agr III |  | Strommenger et al., 2004 |
| agrIV-F  agrIV-R | GCTCAATTAATGCAATAA  ATGGTACTGTAAACATTA | Agr IV |  | Strommenger et al., 2004 |
